# Global Identification of Genes Specific for Rice Meiosis

**DOI:** 10.1371/journal.pone.0137399

**Published:** 2015-09-22

**Authors:** Bingwei Zhang, Meng Xu, Shiquan Bian, Lili Hou, Ding Tang, Yafei Li, Minghong Gu, Zhukuan Cheng, Hengxiu Yu

**Affiliations:** 1 Key Laboratory of Plant Functional Genomics of the Ministry of Education/ Jiangsu Key Laboratory of Crop Genetics and Physiology/Co-Innovation Center for Modem Production Technology of Grain Crops, Yangzhou University, Yangzhou, 225009, China; 2 State Key Laboratory of Plant Genomics and Center for Plant Gene Research, Institute of Genetics and Developmental Biology, Chinese Academy of Sciences, Beijing, 100101, China; National Taiwan University, TAIWAN

## Abstract

The leptotene-zygotene transition is a major step in meiotic progression during which pairing between homologous chromosomes is initiated and double strand breaks occur. *OsAM1*, a homologue of maize *AM1* and *Arabidopsis SWI1*, encodes a protein with a coiled-coil domain in its central region that is required for the leptotene-zygotene transition during rice meiosis. To gain more insight into the role of OsAM1 in rice meiosis and identify additional meiosis-specific genes, we characterized the transcriptomes of young panicles of *Osam1* mutant and wild-type rice plants using RNA-Seq combined with bioinformatic and statistical analyses. As a result, a total of 25,750 and 28,455 genes were expressed in young panicles of wild-type and *Osam1* mutant plants, respectively, and 4,400 differentially expressed genes (DEGs; log_2_ Ratio ≥ 1, FDR ≤ 0.05) were identified. Of these DEGs, four known rice meiosis-specific genes were detected, and 22 new putative meiosis-related genes were found by mapping these DEGs to reference biological pathways in the KEGG database. We identified eight additional well-conserved OsAM1*-*responsive rice meiotic genes by comparing our RNA-Seq data with known meiotic genes in *Arabidopsis* and fission yeast.

## Introduction

Meiosis is a critical process in eukaryotes, occupying a central role in the reproduction and life cycles of all sexually reproducing organisms. Meiosis differs from mitosis in that one round of DNA replication is followed by two sequential cell divisions, leading to the generation of four haploid cells from a single initial diploid cell. The chromosome number of the zygote derived from fertilization recovers to that of the parents [1]. Then, meiosis is important for sexually reproducing organisms to retain the stability of their genetic materials. Moreover, genetic variations can be produced in meiosis through homologous chromosome recombination during prophase I [2].

Despite more than a century of scientific research efforts, the mechanisms underlying meiosis remain largely obscure, particularly with respect to meiosis initiation and the series of events that occur in prophase I, including homologous chromosome recognition, pairing, synapsis and recombination [3–5]. Although an abundance of components controlling the meiotic processes have been identified, little is known about the mechanisms that underlies the initiation of meiosis and phase transitions, especially for plants. Moreover, the molecular mechanisms controlling these events vary greatly among multicellular organisms [6]. In fission yeast, the RNA-binding protein Mei2 is a substrate of Pat1 kinase, and dephosphorylation of Mei2 is sufficient to switch the cell cycle from mitosis to meiosis [7]. In mammals, mouse STRA8 regulates retinoic acid signaling and functions in the initiation of meiosis in female meiocytes [8].

During meiotic prophase I, highly organized processes involving homologous chromosome recognition, alignment, recombination and synapsis promote faithful segregation of homologous chromosomes at anaphase I. According to its chromosomal features and characters, prophase I is divided into five sub-stages: leptotene, zygotene, pachytene, diplotene and diakinesis. Little is known about the transitions between stages. The leptotene to zygotene transition represents a major step in meiotic progression, during which, the initiation of homologous chromosome paring occurs. Throughout this stage, in many organisms, telomeres attach to the inner nuclear membrane and subsequently cluster in a narrow region of the nuclear envelope to form a chromosomal bouquet. As telomeres cluster on the nuclear envelope, double-strand breaks occur, which is proceeded by homologous recombination [1, 9, 10]. In budding yeast, bouquet formation depends on the presence of meiosis-specific telomere proteins Ndj1/Tam1 and actin polymerization [11]. Bouquet formation is believed to facilitate telomere pairing. The movement and juxtapositioning of homologous centromere regions at early zygotene and subsequent synapsis of homologous chromosomes follow this during zygotene and pachytene [12–14]. Therefore, the clustering of telomeres may be one of the possible mechanisms that facilitate initial homology recognition. However, in *Sordaria*, homologs have completed their homology search prior to bouquet formation, meanwhile *phs1* exhibits little effect on bouquet formation in ~50% of the meiocytes [15, 16]. Moreover, *Caenorhabditis elegans* and *Drosophila* both lack bouquets, but can undergo complete meiosis [17].

In plants, a few genes have been reported that function in meiotic initiation and the phase transition. The first mutant in this event, *ameiotic1* (*am1*), was isolated in maize decades ago [18, 19]. *AM1* encodes a plant-specific nuclear protein that is required for meiotic entry and progression through early prophase I. In many maize *am1* mutants, such as *am1-489*, pre-meiotic cells lose their ability to enter meiosis. Instead, these cells undergo several mitotic divisions before they are degraded. In addition, maize *AM1* has a second downstream function, i.e., it participates in regulating the transitional process from leptotene to zygotene during early meiotic prophase I (such as in the mutant *am1-pral*) [18, 20, 21]. Microarray analysis of anther transcriptome modulations by two distinct *am1* alleles, *am1-489* and *am1-pral*, redefines the role of AM1 as a modulator of expression of a subset of meiotic genes [22]. This provides a stage-specific insight into the genetic networks associated with meiotic entry and early prophase I progression. SWITCH1 (SWI1) is the homolog of AM1 in *Arabidopsis*. In *swi1-2* microsporocytes, chromosome arms and centromeres lose their cohesion, resulting in the formation of 20 chromosomes rather than five bivalents at metaphase I. However, the megasporocytes retain centric/whole-arm cohesion but lack synapsis and undergo a mitosis-like division instead of a reductional meiosis [23, 24]. Therefore, although the basic functions of AM1-like proteins in meiosis are well conserved among plants, evolutionary divergence has occurred, even between megasporocytes and microsporocytes in *Arabidopsis*.

Compared with yeast and *Arabidopsis*, little is known about meiotic networks in rice [25], only a few rice genes related to meiosis initiation and phase transition have been identified. *MEL1* (*MEIOSIS ARRESTED AT LEPTOTENE 1*), a germ cell-specific member of ARGONAUTE family, is important in the progression of pre-meiotic mitosis and meiosis in rice [26]. In the *mel1* mutant, meiosis is arrested in early prophase I and chromosomes retain an uncondensed morphology, similar to that during leptotene or zygotene [26]. *MIL1* (*MICROSPORELESS1*) encodes a plant-specific CC-type glutaredoxin, which interacts with TGA transcription factors [27]. In *mil1*, sporogenous cell progenies cannot entry meiosis and anther loculi are filled with somatic cells. MIL1 triggers an anther specific mechanism that directs the meiotic initiation in microsporocytes. *OsAM1*, a homolog of *Arabidopsis SWI1* and maize *AM1*, also encodes a protein with a coiled-coil domain in its central region. But rice AM1 plays an important role in the early meiotic stage transition rather than in meiosis initiation [28]. In the *Osam1* mutant, pollen mother cells are arrested at leptotene, showing that OsAM1 is required for the leptotene-zygotene transition. Che et al. found that only faint OsREC8 signals [29], and no PAIR2 [30], ZEP1[31] or OsMER3 [32] foci, in *Osam1* meiocytes. In contrast, OsAM1 was loaded normally in *pair2*, *Osmer3* and *zep1* meiocytes, suggesting that OsAM1 plays a fundamental role in building stable chromosome structures required for meiosis initiation [28]. However, the exact roles of OsAM1 in leptotene-zygotene transition remain obscure. To gain more insight into the role of OsAM1 in rice meiosis and identify additional meiosis-specific genes, we compared the young panicles transcriptomes of the *Osam1* mutant with wild-type using deep RNA sequencing (the Illumina RNA-Seq method). The results of this study provide a framework for future functional analysis of OsAM1 network in rice meiosis, which might further advance our understanding of the rice meiotic process.

## Material and Methods

### Plant material

The *Osam1* mutant, which was derived from an *indica* variety Zhongxian 3037, was previously induced by ^60^Co~γ ray radiation. Sequence analysis revealed a two-nucleotide deletion in the second exon of *OsAM1*, introducing a premature stop codon [28]. Zhongxian 3037 was used as the wild-type reference in this study. All plants were grown in a paddy field under normal growth conditions at Yangzhou University, China.

### Total RNA isolation, cDNA library preparation and sequencing

Before sample collection, we examined the stage of meiocytes under the microscope. Then we collected spikelets with meiocytes in prophase I of meiosis from panicles that were approximately 40–60 mm in length according to the described criterion [33]. Total RNA samples were extracted from spikelets in meiosis of both the wild-type and the *Osam1* mutant plants using Trizol according to the manufacture’s protocol (Invitrogen, http://www.invitrogen.com). Each sample that underwent RNA extraction included spikelets collected from several panicles. An OligoTex mRNA mini kit (Qiagen) was used to isolate poly (A) mRNA and to prepare a nondirectional Illumina RNA-Seq library with an mRNA-Seq 8 Sample Prep Kit (Illumina). The gel extraction step was modified by dissolving excised gel slices at room temperature to avoid underrepresentation of AT-rich sequences. Purified DNA libraries were amplified by 18 cycles of PCR. The products were loaded onto an Illumina HiSeq2000 instrument and subjected to 100 cycles of paired-end (2×100 bp) sequencing. The raw and processed datasets have been deposited in the Gene Expression Omnibus (GEO) repository (http://www.ncbi.nlm.nih.gov/geo/query/acc.cgi?acc=GSE66469) with an assigned accession number of GSE66469.

### Mapping reads to the reference genome and annotated genes

Rice genome and gene information was downloaded from the Rice Genome Annotation Project (http://rice.plantbiology.msu.edu). Raw image data derived from sequencing were transformed by culling into sequence data. Prior to mapping reads to the reference database, all sequences were filtered to remove adaptor sequences and low-quality sequences (the percentage of low quality bases with a quality value ≤5 was >50% in a read). The remaining reads were aligned to the rice genome using SOAPaligner/soap2, allowing up to two base mismatches.

### Evaluation of genes from RNA-Seq and GO (gene ontology) analysis

ERANGE software (version 4.0) (http://woldlab.caltech.edu/gitweb/) was used to calculate gene expression levels by assigning reads to their site of origin and counting the number of reads. The expression levels of genes from RNA-Seq were normalized by reads per kilo-base per million reads (RPKM) values [34]. For all RPKM values for each gene, the cutoff value for determining gene transcriptional activity was determined based on RPKM value >0. Blast2GO (version 2.3.5; http://www.blast2go.org/) was used to identify each gene with default parameters, and it was also used for GO functional enrichment analysis of certain genes by performing Fisher’s exact test with a robust false discovery rate (FDR) correction to obtain an adjusted p-value between certain test gene groups and whole genome annotation.

### Identification of different expressed genes and pathway analysis

A rigorous algorithm was developed to identify DEGs between two samples based on the number of reads per gene [35]. In the multiple test and analysis, FDR was used to determine the threshold of P values. “FDR < 0.05 and the absolute value of log_2_Ratio ≥ 1” were used as the threshold for judging the significance of differential expression for each gene. For pathway analysis, all significant DEGs were mapped to terms in the KEGG (Kyoto Encyclopedia of Genes and Genomes) database. KEGG pathway analyses were performed using Cytoscape software (version 2.6.2; http://www.cytoscape.org/) with the ClueGO plugin (http://www.ici.upmc.fr/cluego/cluegoDownload.shtml) [36].

### Validation of RNA-Seq by quantitative real-time PCR (qRT-PCR)

For qRT-PCR analysis, total RNA was extracted from spikelets during meiosis of different plants from those used for RNA-seq analysis in the same group (both wild type and mutants). Eleven randomly selected genes together with two genes playing specific functions at early stage of meiosis, *MIL1* (Os07g05630) and *MEL1* (Os03g58600), were inspected. Primer sequences are listed in S1 Table. PCR amplification was performed using SYBR ^**®**^ Premix Ex Taq^TM^ (Takara Bio, Inc.). The amplification protocol was 95°C for 5 min followed by 40 cycles of 95°C for 10 sec, 60°C for 30 sec. Melting curve analysis was performed every 0.5°C from 55 to 95°C with a 10 sec hold at each step to check for the occurrence of a single amplification. Using an ABI StepOne^TM^ Real-Time PCR analysis system (Applied Biosystems), quantification analysis was performed relative to a standard curve according to the cycle threshold values generated. All PCR amplifications were performed three times for each gene and each biological replicate.

## Results

### Transcriptome sequencing

The transcriptomes of young panicles of *Osam1* mutant and wild-type plants were generated, with an average genome coverage of over 10×. As a result, we obtained 51.7 million high-quality reads, each 100 bp in length, encompassing 9 Gb of sequencing data (Table 1). The sequence reads were aligned to the rice reference genome database (Rice Genome Annotation Project) using SOAPaligner/soap2 software (set to allow two base mismatches). Of these reads, 84.05% mapped to unique (49.16%) or multiple (34.89%) genomic locations; the expression levels of all unique transcripts that were mapped to the genome were quantified as RPKM values [34]. On the other hand, 15.95% of the sequence reads had no match in the reference genome. Using this technique, only reads aligning entirely inside exonic regions can be matched to the transcripts (reads from exon-exon junction regions cannot be match). A comparative analysis of sequencing datasets against Rice Genome Annotation Project reference data, at a cutoff of one read per million reads, revealed that 25,750 genes were expressed in the wild type, while 28,455 genes were detected in the *Osam1* mutant.

**Table 1:** Results of RNA-Seq read mapping.

	Wild-type	*Osam1* mutant
Total clean reads	22,350,753	29,411,589
Mapped reads	18,863,502	24,618,283
	84.40%	83.70%
Unique match	9,700,496	16,155,932
	43.40%	54.93%
Multi-position match	9,163,006	8,462,351
	39.00%	28.77%
Unmapped reads	5,688,271	10,024,052
	15.60%	16.30%

**Fig 1:**
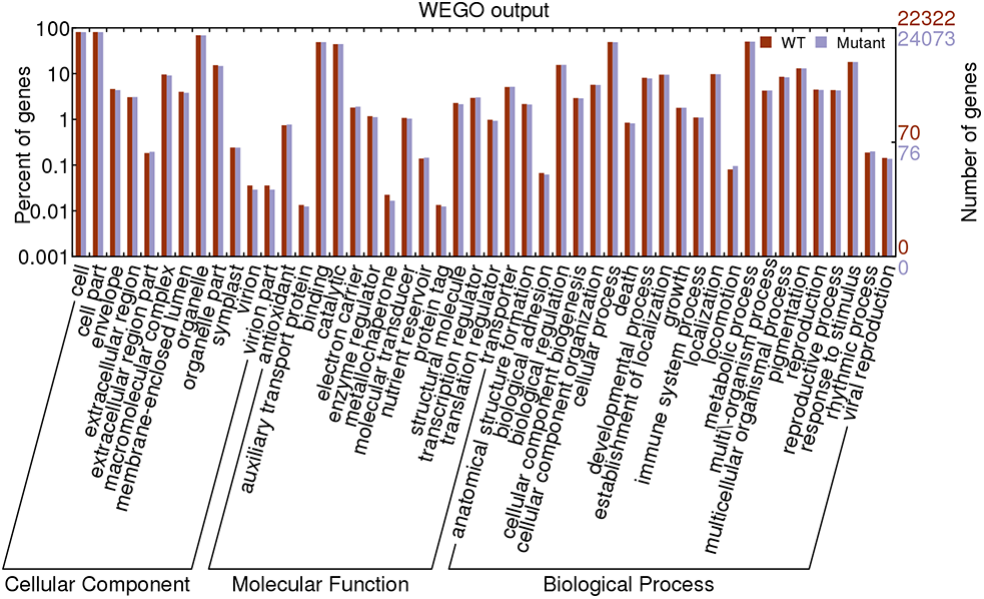
GO classification of all genes between the wild type and *am1* mutant.

To facilitate the global analysis of gene expression, all predicted rice genes were classified into three major groups (biological process, molecular function and cellular component) using Blast2GO (version 2.3.5) (http://www.blast2go.org/). A total of 19,528 GO (gene ontology) terms were associated with all 34,314 expressed genes in the wild type and *Osam1* mutant, which were classified into 48 functional subcategory annotations, providing an overview of ontology content (Fig 1). The top six most abundant sub-groups were cell, cell part, metabolic process, cellular process, organelle part and binding. The GO annotation assignment denoted that the genes expressed in this study encode diverse functional proteins. Notably, the number of genes expressed in the organelle part of cellular component category, reproductive process of biological process category and nucleotide binding category of molecular function category was significantly higher in the *Osam1* mutant than in the wild type.

### Validation by qRT-PCR

**Fig 2:**
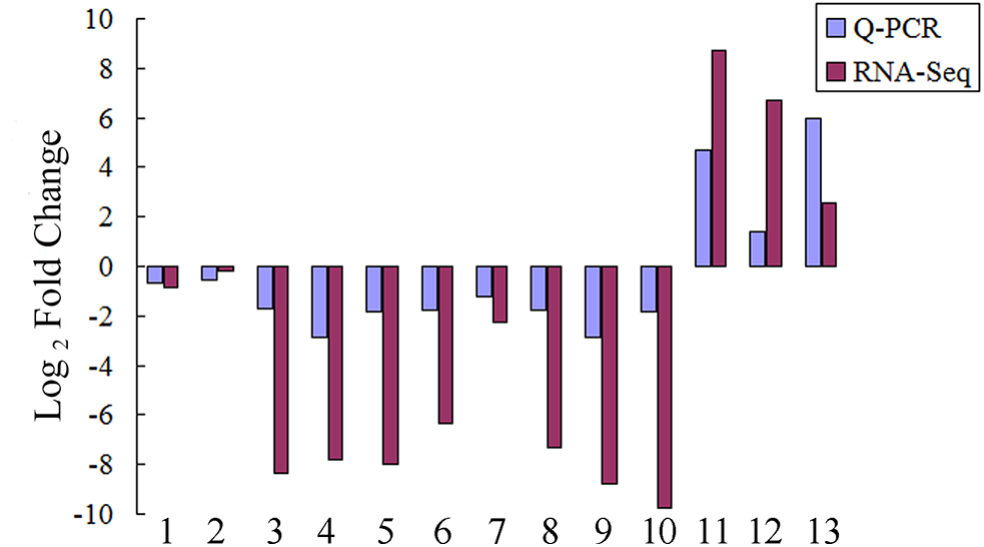
Comparison of expression levels of selected genes estimated by RNA-seq and RT-PCR. Y-axis: log_2_ of estimated fold change between *Osam1* and wild type. X- axis: tested genes, 1, Os07g05630; 2, Os03g58600; 3, Os04g58630; 4, Os08g02380; 5, Os01g49010; 6, Os03g44760; 7, Os02g04080; 8, Os01g50616; 9, Os05g05720; 10, Os09g07510; 11, Os02g53120; 12, Os02g07240; 13, Os03g57310.

To further verify the results of RNA-Seq analysis, eleven randomly selected genes together with two genes performing a specific function at early stage of meiosis, *MIL1* (Os07g05630) and *MEL1* (Os03g58600), were inspected by qRT-PCR in another pair of wild-type and the *Osam1* mutant samples as a partial replicate of RNA-Seq analysis. We compared qRT-PCR results with those generated from RNA-Seq analysis, and found that the expression patterns were consistent between the two experiments for all the genes examined (Fig 2). The qRT-PCR inspection also validates the results of RNA-Seq analysis.

### Identification of DEGs by RNA-Seq

**Fig 3:**
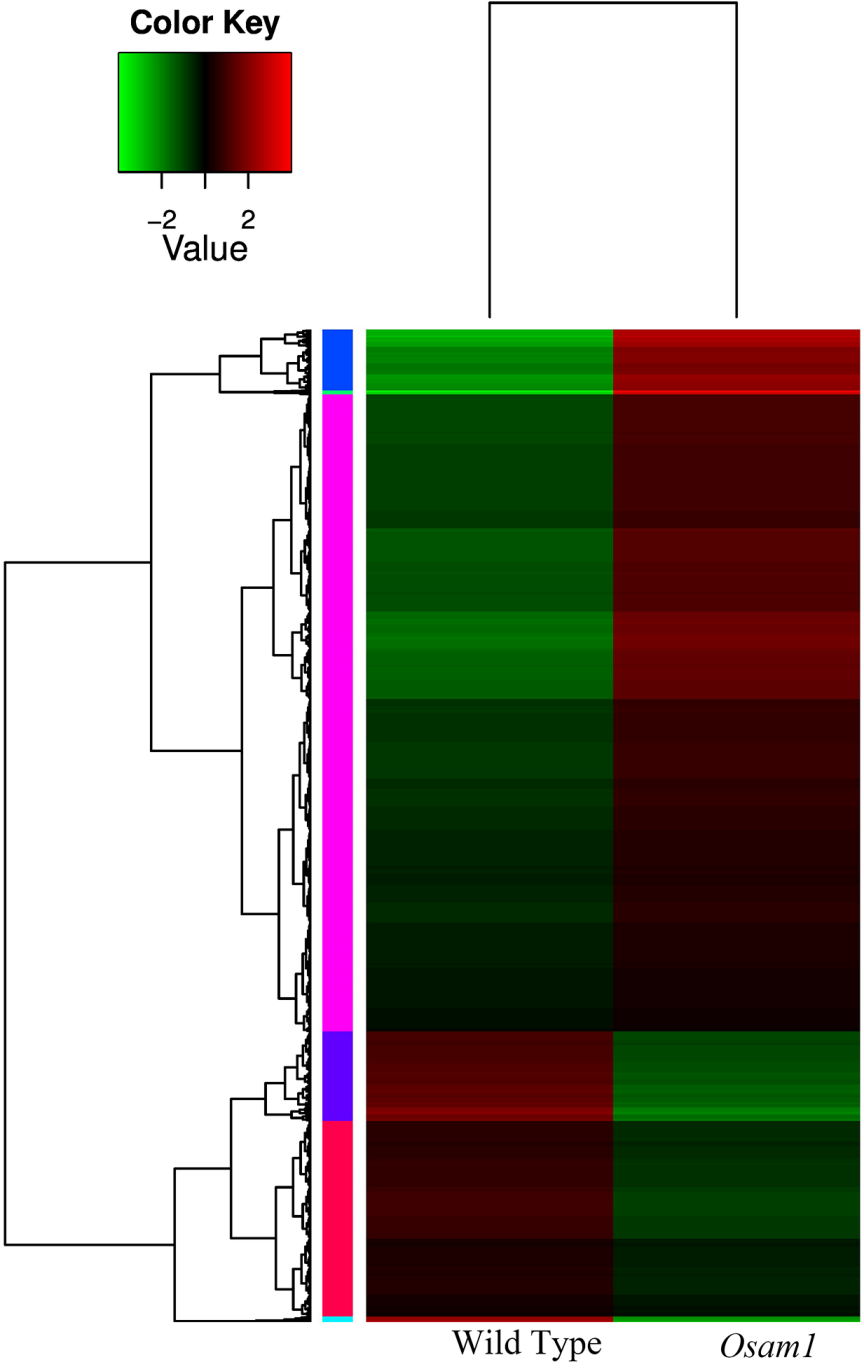
Heatmap of differential expression genes between wild type and *Osam1* plants. Green represents low expression, red represents high expression, each row represents a differentially expressed gene and each column represents a sample. Changes in expression levels are shown in the color scales.

Gene expression levels were calculated as RPKM, with RPKM values ranging from 0 to over 10^4^. RNA-Seq analysis is performed to analyze DEGs between samples, with the goal of obtaining a deeper understanding of these genes. Putative DEGs were identified using the following criteria: (1) false discovery rate less than or equal to 0.05 and (2) fold change (FC) greater than or equal to 2. Using these criteria, a total of 4,400 genes with significantly altered expression levels were detected in the *Osam1* mutant compared to the wild type. Among these genes, 3,012 were up-regulated and 1,388 were down-regulated in the *Osam1* transcriptome compared to the wild type. Using two-dimensional hierarchical clustering, we classified the 4,400 differential expression profiles into six expression cluster groups based on their similarity (Fig 3); separate plots are shown for each cluster, and the means and standard deviations of RPKM expression values are indicated. Groups of genes with similar expression patterns may play the same roles in panicle development.

### KEGG enrichment analysis of DEGs

**Fig 4:**
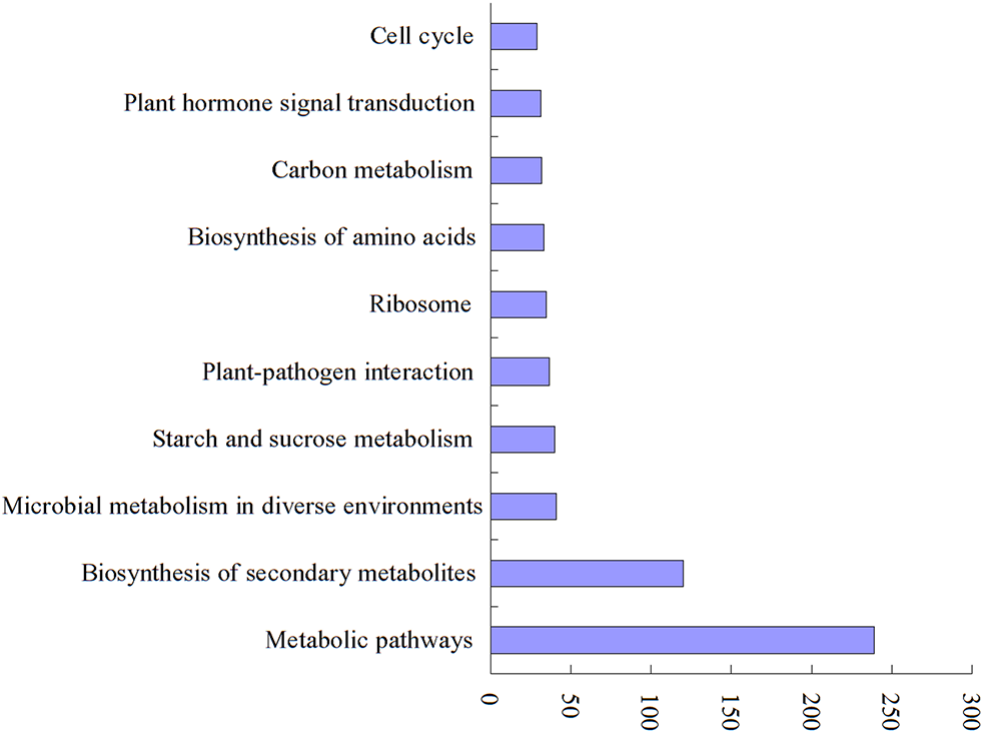
Classification of all DEGs based on KEGG categorization. The top 10 most highly represented categories and the number of transcripts predicted to belong to each category are shown.

The interaction between genes plays an essential role in biological functions. Pathway-based enrichment analysis helps further to elucidate the biological functions of DEGs. KEGG is an important public pathway-related database that integrates genomic, chemical and systemic functional information, providing classifications that are valuable for studies of genetically and biologically complex processes. To perform functional classification and pathway assignment of all DEGs, we performed pathway-based analysis using the KEGG pathway database. As a result, 931 of 4,400 DEGs were classified into 251 KEGG pathways. Of these 931 DEGs, 637 were classified into the top 10 most highly represented categories (Fig 4). Most unigenes were categorized into metabolic pathways (239, 25.67%), biosynthesis of secondary metabolites (120, 12.89%), microbial metabolism in diverse environments (41, 4.40%) and starch and sucrose metabolism (40, 4.29%) (Fig 4). Those results suggest that there are considerable differences in various physiological processes between young panicles of wild-type and *Osam1* mutant. These annotations represent a valuable resource for investigating specific processes and performing functional analysis.

### Identification of meiosis-preferential genes responsive to *OsAM1*

The meiotic process in the *Osam1* mutant appears to initiate properly, but progression halts in leptotene, which is crucial for homologous chromosome pairing and recombination. Some important events are profoundly affected in the *Osam1* mutant, including sister chromatid cohesion and telomere bouquet formation, as well as homologous recombination and synapsis. We identified meiosis-specific genes that respond to OsAM1 effectively based on two criteria: (1) the genes are DEGs (described above); (2) the genes are involved in meiosis process pathways (as determined by KEGG analysis) or previously identified as rice meiotic genes or rice homologs of meiotic genes in other species. As a result, we identified 22 significant DEGs with functions related to meiotic processes using KEGG pathway analysis, such as genes in the pathways “Meiosis-yeast”, “Oocyte-meiosis”, “Homologous recombination”, “DNA replication”, “Nucleotide excision repair” and “Mismatch repair” (Table 2 and Table 3). For example, Os07g22680, a homolog of *Arabidopsis SKP1-LIKE* genes in rice, was classified into the “Oocyte-meiosis” pathway. In *Arabidopsis*, *SKP1-LIKE1* (*ASK1*) plays a critical role in many cellular processes including meiotic chromatin reconstruction, telomere formation and recombination [37, 38]. Os01g49010 and Os12g03130 were classified into meiosis-yeast and homology to *ORC4* and *CDC4*, respectively; these genes play crucial roles in DNA replication [39, 40]. These results indicate that the genes involved in these pathways have putative functions in meiosis.

**Table 2:** List of significantly DEGs related to meiosis identified by KEGG pathway analysis.

Pathway name	Gene ID	Protein feature	Differently expressed
Meiosis-Yeast	LOC_Os09g07510	HEAT repeat family protein	Down
	LOC_Os03g07150	male sterility protein, putative, expressed	Down
	LOC_Os03g16110	Ser/Thr protein phosphatase family protein, putative, expressed	Down
	LOC_Os03g59060	OsPP2Ac-2—Phosphatase 2A isoform 2 belonging to family 2, expressed	Up
	LOC_Os02g47180	WD repeat-containing protein, putative, expressed	Down
	LOC_Os05g05720	tetratricopeptide repeat domain containing protein, expressed	Down
	LOC_Os02g53120	Peptidase family C50, putative, expressed	Up
	LOC_Os12g43120	expressed protein	Down
	LOC_Os01g49010	ORC4—Putative origin recognition complex subunit 4, expressed	Down
	LOC_Os12g03130	CDC45A - Putative DNA replication initiation protein, expressed	Down
	LOC_Os05g50360	anaphase-promoting complex subunit 10, putative, expressed	Down
DNA Replication	LOC_Os02g07240	expressed protein	Up
Homologous Recombination	LOC_Os03g06920	DRD1, putative, expressed	Up
	LOC_Os01g14980	RPA3—Putative single-stranded DNA binding complex subunit 3, expressed	Up
Nucleotide Excision Repair	LOC_Os08g33340	RAD23 DNA repair protein, putative, expressed	Up
	LOC_Os06g07480	CDK-activating kinase assembly factor	Down
Mismatch Repair	LOC_Os04g58630	DNA mismatch repair protein MSH3, putative, expressed	Down
	LOC_Os01g56940	flap endonuclease, putative, expressed	Up

**Table 3:** List of significant DEGs related to meiosis identified by KEGG pathway analysis.

Pathway name	Gene ID	Protein feature	Differentlyexpressed
Oocyte Meiosis	LOC_Os03g17700	CGMC_MAPKCGMC_2_ERK.2 - CGMC includes CDA, MAPK, GSK3, and CLKC kinases, expressed	Up
	LOC_Os02g36974	14-3-3 protein, putative, expressed	Down
	LOC_Os07g22680	SKP1-like protein 1B, putative, expressed	Up
	LOC_Os03g02680	cyclin-dependent kinase A-1, putative, expressed	Down

There are 33 known meiosis-associated genes in rice [41–44]. We investigated the expression levels of these genes to identify OsAM1-responsive genes. Among these 33 genes, four were found to be significant DEGs between the *Osam1* mutant and wild type, including *OsHEI10* [45], *OsMSH5* [46], *OsZIP4* [47] and *PSS1* [48].

To further identify putative meiosis-related genes that respond to OsAM1, we used INPARANOID software [49] to identify putative rice orthologs among these significant DEGs according to known meiosis-related genes in other organisms, including *Arabidopsis* and fission yeast (*Schizosaccharomyces pombe*) [50, 51]. As a result, eight additional well-conserved rice meiotic genes that respond to OsAM1 were identified (Table 4). Of these eight meiotic genes, five are homologous to *Arabidopsis* genes *SMC3* [52], *ATR* [53], *ATM* [54], *RMI1* [55] and *MPA1*[56], respectively (Table 4). The remaining three meiotic genes are homologous to *PSY1* [57], *MEU22* [58] and *SPO20* [59] in fission yeast, respectively (Table 4).

**Table 4:** List of significant DEGs homologous to known meiotic genes in *Arabidopsis* and budding yeast.

Gene ID	Homolog	Description for rice genes	Differently expressed
LOC_Os06g50910	ATR	protein Phosphatidylinositol kinase and FAT containing domain protein, putative, expressed	Up
LOC_Os01g01689	ATM	protein phosphatidylinositol 3- and 4-kinase family protein, expressed	Up
LOC_Os04g32090	RMI1	protein expressed protein	Down
LOC_Os08g44860	MPA1	protein aminopeptidase, putative, expressed	Up
LOC_Os02g04080	SMC3	protein chromosome segregation protein sudA, putative	Down
LOC_Os03g45170	MEU22	protein amino acid permease, putative, expressed	Up
LOC_Os03g57310	PSY1	protein syntaxin, putative, expressed	Up
LOC_Os01g50616	SPO20	protein phosphatidylinositol transfer, putative, expressed	down

## Discussion

In plants, meiocytes differentiate late in floral ontogeny. The mechanisms underlying meiocyte differentiation from floral somatic cells are currently unknown. However, nuclear events in meiotic cells are readily distinguishable from those in mitotic cells, and the developmental program of archesporial cells and meiocytes is independent of successive somatic cell development, at least for the progression into prophase I [60]. In the *Osam1* mutant, pollen mother cells are arrested at leptotene, and several meiotic processes are affected, including sister chromatid cohesion, telomere bouquet formation, homology search, homologous recombination and synapsis. In the current study, global transcriptome analysis reinforced the distinctions between wild-type and *Osam1* plants. Using RNA-Seq analysis, many known or putative meiosis-related, OsAM1-responsive genes were identified, including four known rice meiosis-specific genes, i.e., *OsHEI10*, *OsMSH5*, *OsZIP4* and *PSS1*. These results further strengthen the notion that OsAM1 plays a role in modulating the expression of many critical meiotic genes. While several other important genes, including *PAIR2*, *ZEP1* and *OsMER3*, were expressed at similar levels in *Osam1* and the wild type, the corresponding proteins were not detected by immunostaining in *Osam1* meiocytes [28]. This result indicates that although *PAIR2*, *ZEP1* and *OsMER3* can be expressed in *Osam1*, the corresponding proteins fail to load onto chromosomes normally. These results imply that these three genes are not essential for the leptotene-zygotene transition in rice, but OsAM1 is required for their chromosome localization.

Most maize *am1* mutants cannot enter meiosis. Only *am1-praI* can enter meiosis, but meiosis progression stops in leptotene [18, 20, 21]. Two rice *am1* mutants and RNAi lines enter meiosis, but progression halts in leptotene. The chromosome behaviors of rice *Osam1* and maize *am1-praI* appear to be similar, but some differences exist between these lines. In maize *am1-praI* meiocytes, punctuate ASY1 signals and some continuous stretches were revealed by immunostaining, indicating partial formation of the chromosome axis, which was not the case in rice. Also, in maize *am1-praI* meiocytes, strong REC8 signals were observed, which partially localized to chromatin, but only faint OsREC8 foci were observed in *Osam1* meiocytes [18, 28]. Thus, AM1-like proteins may exhibit functional variance, even among monocots. In the present study, four known meiosis-related genes, including *OsHEI10* [45], *OsMSH5* [46], *OsZIP4* [47] and *PSS1* [48], were differentially expressed in *Osam1* compared to the wild type. Of these four genes, the expression of *ZIP4* was down regulated in *Osam1*, which is consistent with the change observed in the maize *am1-praI* mutant. The expression level of *MSH5* was up regulated in *Osam1*, but this gene is not significantly differentially expressed in *am1-praI* based on oligonucleotide GeneChip array analysis [61]. In the current study, several other well-known meiotic genes in rice, such as *SPO11-1* [62], *REC8* [29], *PAIR1* [63] and *RAD51* [64], were not differentially expressed in the *Osam1* mutant compared to the wild type, which is in agreement with the results obtained for maize *am1-praI*. These results shed light on the conservation of the leptotene-zygotene transition process between rice and maize.

The leptotene-zygotene transition is critical in many aspects, including active and passive chromosome movements, at least in some organisms [1]. Several meiosis-associated transcript changes were identified in our transcriptome profiling study, which may play a major role in rice leptotene-zygotene transition. *OSK21* (Os07g22680), a putative meiotic gene belonging to the *SKP1* (S-phase Kinase-Associated Protein1) gene family is up-regulated in the *Osam1* mutant. *SKP1* plays a key role in cell-cycle progression, transcriptional regulation, signal transduction and many other cellular processes in eukaryotes [65]. In *Arabidopsis SKP1-LIKE1* (*ASK1*) gene plays a critical role in recombination during the leptotene to pachytene transition [37, 38]. ASK1 protein is also important for many cell processes including reconstructing meiotic chromatin and telomere formation [25]. Therefore, *OSK21* may play an important role in the leptotene-zygotene transition in rice meiosis. We also found some other genes (Os01g14980, Os06g50910, Os01g49010 and Os12g03130) showed significant expression changes in the *Osam1* mutants compared with the fertile plants. They were homologs of *RPA3*, ATR, *ORC4* and *CDC4*5, respectively. These genes play critical roles at interphase when cells are preparing for meiotic cell cycle, including cell growth, DNA replication in *Arabidopsis* and fission yeast [39, 40, 53, 66]. Therefore, we speculate that a switch in cell processes programmed by regulating gene expression at interphase is necessary for subsequent leptotene-zygotene transition and normal development of archesporial cells.

Meiocytes that have been isolated using manual methods or laser capture microdissection are relatively free of contamination of somatic cells. Multiple microarray and RNA-Seq analyses of purified meiocytes have recently been reported [50, 67–70]. In the present study, we subjected spikelets of young panicles to deep RNA-sequencing. Although spikelets of young panicles contain many somatic cells, we were able to identify some significant DEGs by comparing the transcriptomes of *Osam1* mutant and the wild-type. We also validated the transcription patterns of several meiosis-specific genes using qRT-PCR. The expression patterns were consistent between two analyses for all transcripts tested. Nevertheless, it is possible that there were false negatives due to a failure of the fold-change method. We observed two-fold changes in gene expression for numerous genes. However, for genes with higher expression levels, smaller changes in gene expression may have occurred, but these changes may have been rejected by the fold-change method. Thus, we may have failed to detect some OsAM1-responsive, meiosis-specific genes.

## Conclusions

Meiosis is an essential step in sexual reproduction and one of the most ancient processes that facilitate the redistribution of genetic variation and increase biodiversity. However, our understanding of rice meiosis is limited, as only a small number meiosis-specific gene functions have been identified. Our analysis revealed large differences in the transcriptomes of young panicles of *Osam1* compared to the wild type. The expression levels of many meiosis-associated genes were highly altered in the *Osam1* mutant, including genes important for DNA replication, synapsis, homologous recombination and other critical events. The functions of many of these genes in rice remain uncharacterized. These findings provide a framework for future functional analysis of genes involved in the rice leptotene-zygotene transition process and advance our understanding of rice meiosis.

## Supporting Information

S1 Table List of primers used in quantitative real-time PCR. (XLS)
